# Devitalizing noise-driven instability of entangling logic in silicon devices with bias controls

**DOI:** 10.1038/s41598-022-19404-0

**Published:** 2022-09-07

**Authors:** Hoon Ryu, Ji-Hoon Kang

**Affiliations:** Korea Institute of Science and Technology Information, Daejeon, 34141 Republic of Korea

**Keywords:** Engineering, Nanoscience and technology, Physics

## Abstract

The quality of quantum bits (qubits) in silicon is highly vulnerable to charge noise that is omnipresent in semiconductor devices and is in principle hard to be suppressed. For a realistically sized quantum dot system based on a silicon-germanium heterostructure whose confinement is manipulated with electrical biases imposed on top electrodes, we computationally explore the noise-robustness of 2-qubit entangling operations with a focus on the controlled-X (CNOT) logic that is essential for designs of gate-based universal quantum logic circuits. With device simulations based on the physics of bulk semiconductors augmented with electronic structure calculations, we not only quantify the degradation in fidelity of single-step CNOT operations with respect to the strength of charge noise, but also discuss a strategy of device engineering that can significantly enhance noise-robustness of CNOT operations with almost no sacrifice of speed compared to the single-step case. Details of device designs and controls that this work presents can establish practical guideline for potential efforts to secure silicon-based quantum processors using an electrode-driven quantum dot platform.

## Introduction

The spin of electrons in isotopically enriched silicon (Si) has been regarded as a promising mechanism for encoding quantum information due to its extremely long coherence time^1–4^ that is highly advantageous for stable manipulations of quantum bits (qubits). In particular, a great amount of effort has been put in by researchers to physically realize universal logic gate devices with electron spins in Si-based quantum dot (QD) structures^3–16^ whose confinement is controlled with external electric and magnetic fields. The preciseness of corresponding logic operations has been continuously improved so single qubit rotations can be now conducted with a fidelity larger than 99%^3–7,11,13–16^, and recently two-qubit entangling operations with a high fidelity are also reported, e.g., 2-qubit SWAP operations with a 98% fidelity^8^, 2-qubit controlled-Z (CZ) operations with a fidelity larger than 99%^12,14^ and 2-qubit synthesized controlled-X (CNOT) operations with a 98.6% fidelity^13^. Elaborated designs of gate devices that generate quantum entanglement^17,18^, the most celebrated quantum resource being widely used in various applications^19–21^, have been also reported but their accuracy so far is generally not as good as the non-entangling cases so the fidelity of 2-qubit Bell-states generated from double quantum dot (DQD) platforms stays in 78–97%^9–11,13,15^. With a rapid progress in pulsing technologies^6,22^, the speed of DQD-based gating operations reached a sub-microsecond level, and it is shown that a CNOT operation, one of most crucial entangling logics for universal quantum computing, can be conducted in less than 200 nanoseconds (ns) with a single microwave pulse^10^.

In general, the quality of spin qubits in solid-based platforms highly depends on material-inherent noises^23–27^ that are mainly due to the fluctuation in local electric and magnetic fields around qubits. In the Si-based case, noise of magnetic fields (spin noise) can be suppressed with purification of $^{28}$Si crystals, and latest works have shown 12-inch $^{28}$Si wafers that contain 100 ppm or less of spin-carrying $^{29}$Si atoms^28,29^. Suppressing noise of electric fields (charge noise), however, is more difficult than the case of spin noise since its origins have not been fully understood yet. Accordingly, state-of-the-art ideas have been proposed to increase the robustness of spin qubits to existing charge noises in Si devices such as, for example, placing qubits far away from surface oxides^27^ that can serve as a source of low-frequency charge noises^24^, increasing spin resonance frequencies^7^, biasing DQDs symmetrically to reduce the sensitivity of qubit interactions to charge noises^10,12,30^, and decomposing a CZ gate into two CZ/2 gates bridged by an X gate which decouples the quasi-static single-qubit phase noise^14^. Theoretical concepts have been also suggested for the robustness of entangling gates to charge noise, e.g., using ultra-fast Rydberg interaction for entangling gate in silicon donor^31^ and optimizing pulse sequences by a neural network to compensate crosstalk of signals under the existence of charge noise^32^. In spite of the non-trivial contribution driven with these ideas, the up-to-date fidelity of entangling operations in Si devices is not yet in a level where the accuracy in computations can be generally guaranteed, and the motivation for sound studies on technical strategies that can enhance the fidelity of entangling operations under charge noise, therefore, should be huge.

In this work, we elaborately examine the engineering-driven possibilities for devitalizing negative effects that charge noise have against entangling operations implemented with Si QD devices, where the focal point of engineering is the real-time pattern of control signals that has been rarely discussed in detail by the strategies proposed in previous studies^7,27,30^. For this purpose, we computationally explore Si DQD structures with our in-house simulation code package that can describe device operations in a full-scale from initializations and time-dependent behaviors of electron spin qubits. As a baseline for discussion, we first model the fast CNOT operation that is driven with a single-step pulse in the recently reported DQD platform^10^, and quantify fluctuations in fidelity under charge noise by incorporating random noisy potential profiles to device simulations as charge noise can be defined as fluctuations of electric potential energy^30,33^. Then, as an alternative way, we implement a CNOT operation with a multi-step control that does not employ AC microwave pulses for generation of entanglement. In spite of the loss in fidelity that happens during the real-time transition of control signals, we find a general pattern that the resulting CNOT operation has remarkably increased robustness to charge noise whilst its operating speed can be maintained in a same order compared to the case of a single-step control. Additional in-depth discussion is presented via rigorous modeling to study the optimal control of multi-step CNOT operations in realistic conditions with a trade-off between the speed and the noise-robustness of operations. Being carried as an extension of our preliminary study that focused on the noise-free addressing of individual qubits^34^, this work can make a meaningful contribution for Si-based designs of entangling logic blocks that are essential for development of programmable quantum processors.

## Methods: model problem and simulations

**Figure 1:**
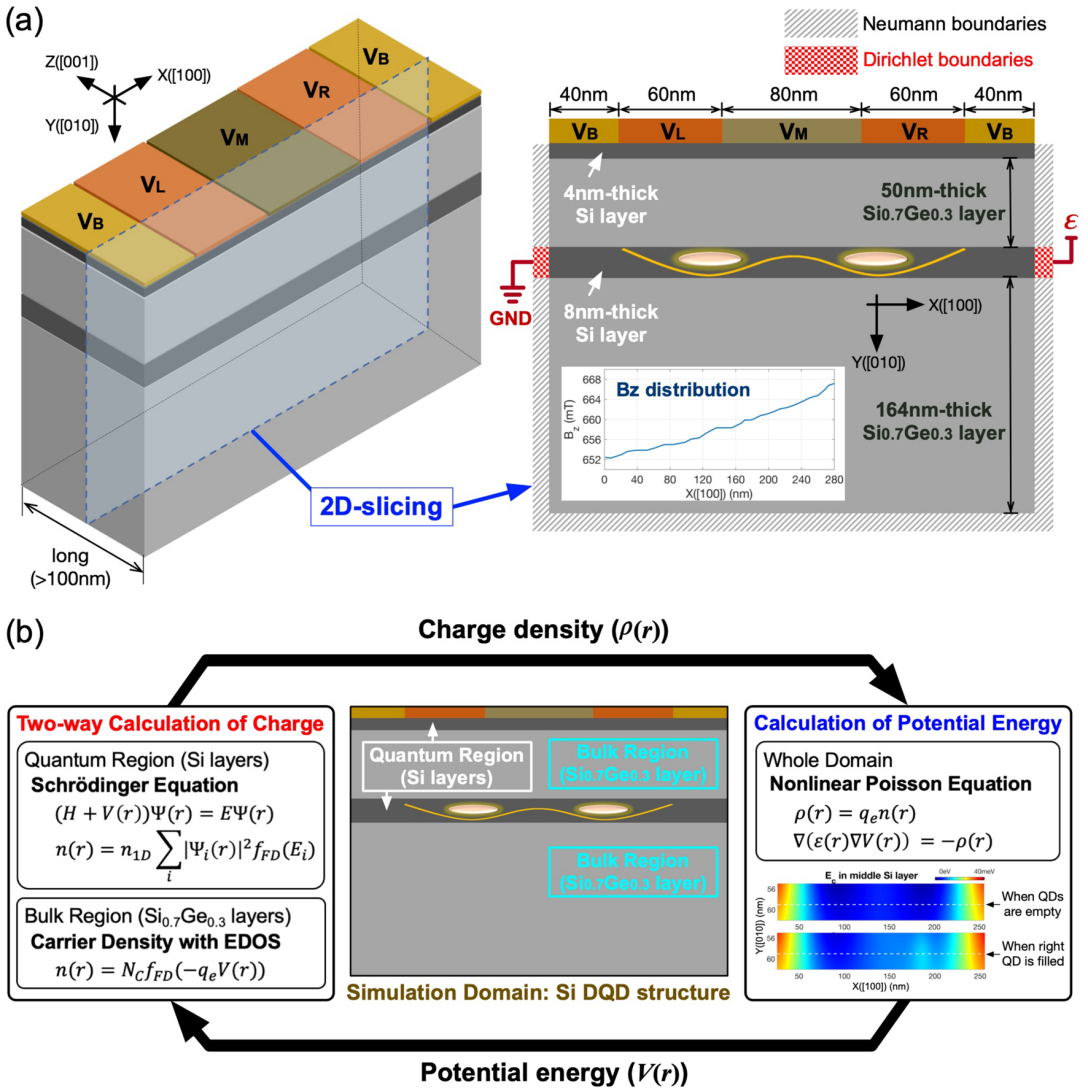
Target structure and multi-scale scheme of device modeling. (**a**) A 3D view of the silicon (Si) double quantum dot (DQD) structure that resembles the physical one reported by Zajac et al. (Ref.^10^). Here the lateral confinement (along the [100] direction) is controlled with DC biases imposed on the top electrodes, while the vertical one (along the [010] direction) is naturally formed due to the band offset among silicon-germanium (SiGe) and Si layers. Since the structure is very long along the [001] direction, we use its 2D slice for device simulations assuming it is infinitely long along that direction (a lateral distribution of the static magnetic field $B_Z$, generated from a horseshoe-shaped cobalt micromagnet, is shown in the inset). (**b**) The self-consistent loop of device simulations used to model spatial distributions of charge and potential. Here the charge distribution at a given potential distribution is obtained in two ways; the electronic structure simulation based on a parabolic effective mass model is used to get the density in Si layers where most of electrons reside, while the region of SiGe layers is treated with the physics of bulk semiconductors.

Figure 1a shows the DQD structure that is adopted as a target of modeling in this work. Mimicking the reported physical system^10^, the target platform is based on a heterostructure that consists of 2 Si and 2 silicon-germanium (SiGe) layers where the fraction of Ge in SiGe layers is 30%. Due to the Si/SiGe band offset, the structure has a natural quantum well along the vertical ([010]) direction and electrons can be confined in the 8 nanometer(nm)-thick Si layer. The lateral ([100]) confinement in the 8nm-thick Si layer is controlled with DC biases imposed on top Ti/Au electrodes (2 barrier gate biases ($V_B$), 1 left/middle/right gate bias ($V_L/V_M/V_R$)), so the system can have up to 2 potential valleys. As the DQD system is quite long (>100nm) along the [001] direction, we use its 2D-slice as a simulation domain assuming the structure is infinitely long along that direction. The top electrodes are considered in device simulations by imposing a Dirichlet boundary condition on a 2D Poisson equation with applied biases and Schottky barrier heights ($\Phi _B$) that are calculated using the work-function reported for Ti/Au metal layers^35^. The source and drain electron reservoirs, which are secured with 2D electron gas (2DEGs) in reality, are also described with Dirichlet boundaries (the two red boundaries in Fig. 1a) where we set $\Phi _B$ to zero assuming that 2DEGs are formed well and are therefore perfectly ohmic. For simulations, we grounded the source and imposed an extremely small bias ($\varepsilon$ $\simeq$ 0.1 mV) on the drain, and a low temperature of 1.5K is assumed.

**Figure 2:**
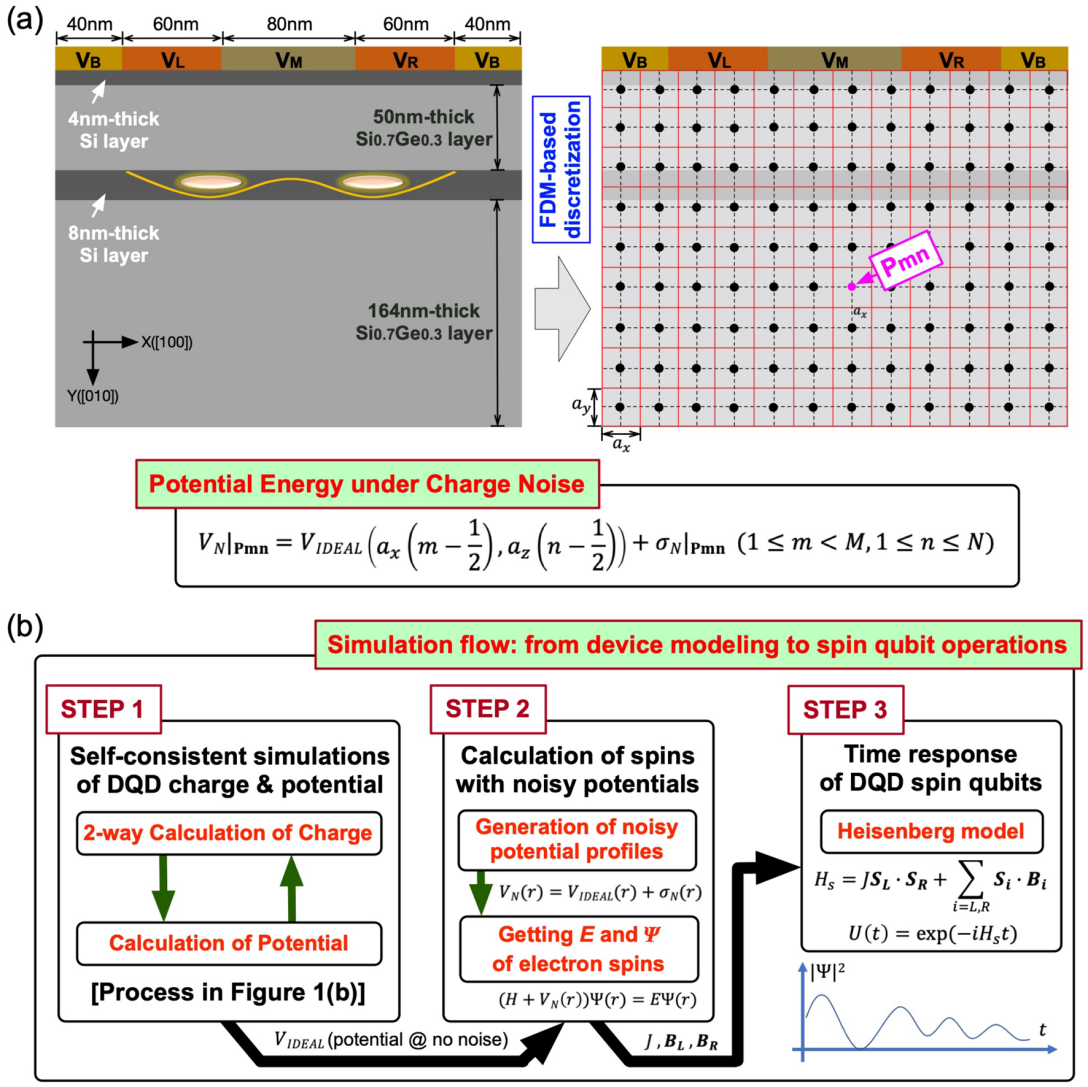
Incorporation of charge noise and steps for full-stack simulations covering from device modeling to qubit operations. (**a**) The effect of charge noise is incorporated into device simulations by disturbing the spatial distribution of potential energy under no noise ($V_{IDEAL}$) with a random value that is generated per real-space grid in a silicon (Si) double quantum dot (DQD) structure under a zero-mean gaussian distribution of a standard deviation up to 5 µeV. (**b**) Starting with device modeling, our full-stack simulation can eventually predict 2-qubit operations of a Si DQD system with the following three steps: (1) device simulations that give bias-dependent energetic positions and wavefunctions of electron spin states under no charge noise, (2) disturbing the noise-free potential profile with charge noise, and (3) solving a time-dependent Schrödinger equation for the Heisenberg Hamiltonian of two neighbor spins that is constructed with results of device simulations.

The spatial distribution of potential energy and electron density in the DQD system, which is the outcome of device simulations, is determined with a self-consistent process described in Fig. 1b. While the potential profile is calculated with a normal Poisson solver, the charge profile is evaluated in two ways with regional dependence so the region of thin Si layers (labeled as **Quantum Region**), which has most of electrons and must be solved quantum mechanically, is treated with electronic structure simulations coupled to a parabolic effective mass model^36^, and the region of SiGe layers (labeled as **Bulk Region**) is solved with the physics of bulk semi-conductors. For precise modeling of spin states, the electronic structure is calculated with a lateral distribution of the static magnetic field along the [001] direction ($B_Z$) that is reported by Neumann et al.^37^ with simulations of the horseshoe-shaped micromagnet employed in the real experiments^10,38,39^ (see the inset of Fig. 1a). Once the potential distribution at a certain set of biases is determined, we disturb this “clean” solution with a noisy potential profile, which is obtained with values that are randomly generated per each real-space grid of the simulation domain as described in Fig. 2a. All the random values here are generated under a zero-mean gaussian distribution, and its standard deviation $\sigma$, which represents the strength of charge noise, is considered up to 5 µeV that is normal in Si-based devices these days^40–43^. Once the ground states of two QDs are known from device simulations, we can construct the Heisenberg 2-spin Hamiltonian with their Zeeman-spitting energies and exchange interaction^33^, and 2-qubit time responses of the DQD system can be then calculated as described in Fig. 2b that shows the scheme of our full-stack modeling.

## Results and discussion

**Figure 3:**
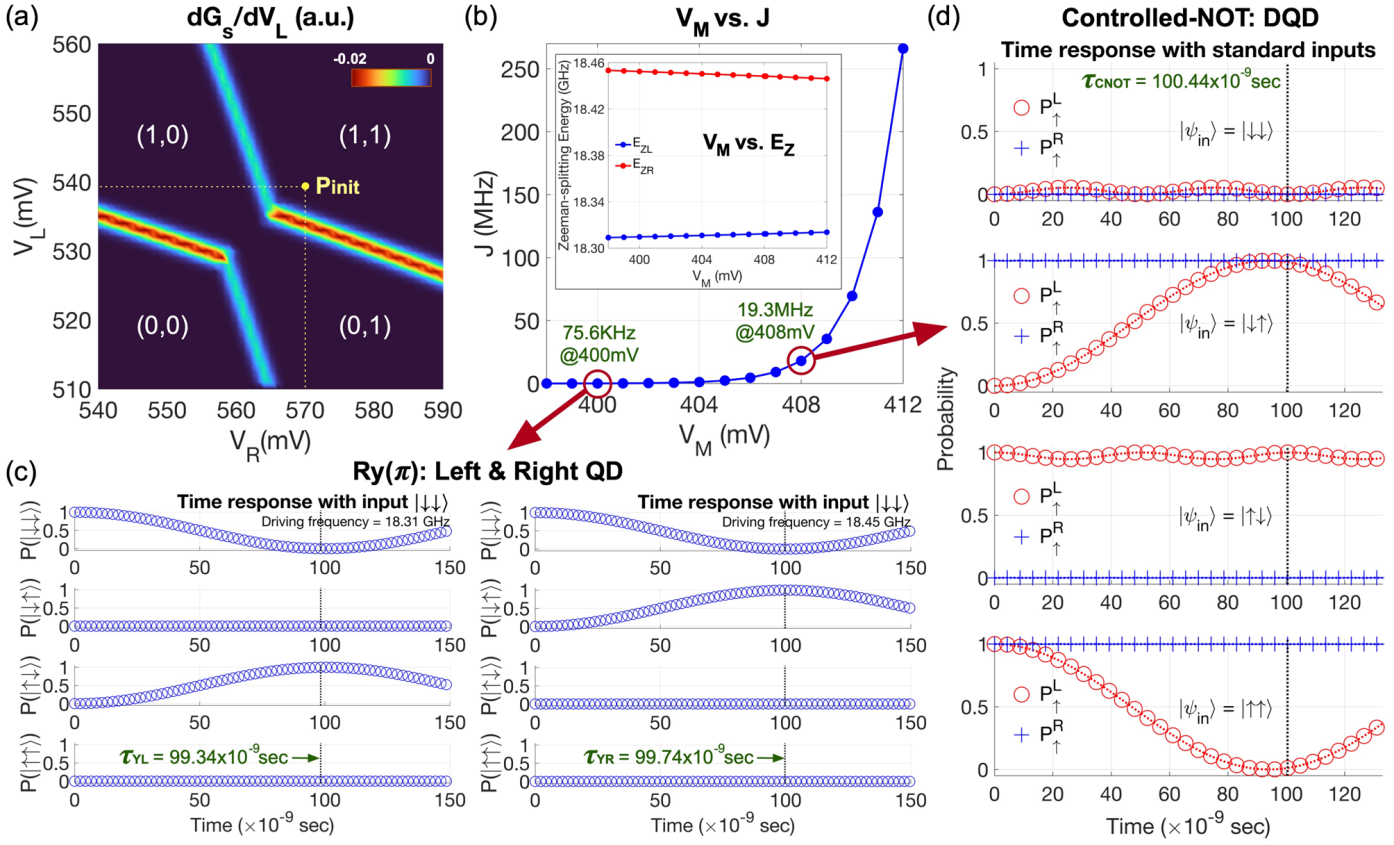
Device initialization, 1-qubit rotation and single-step CNOT operation. (**a**) A charge stability diagram of the double quantum dot (DQD) system that shows electron-filling in each QD as a function of the left & right gate bias ($V_L$ & $V_R$). The middle gate bias ($V_M$) is set to 400 mV. With controls of gate biases, we can fill a single electron in each QD, initializing the device to a $|\downarrow \downarrow \rangle$ state. (**b**) Exchange (*J*) and Zeeman-splitting energy ($E_{ZL}$, $E_{ZR}$) of two QDs shown as a function of $V_M$ when $V_L$ and $V_R$ are 540 mV and 570 mV, respectively. Increasing $V_M$ lowers the potential barrier between two QDs and enhances the interaction between electrons that occupy the down-spin ground state of each QD. (**c**) Time responses of the DQD system at $V_M$ = 400 mV. The $R_Y(\pi )$ operation for the left and right qubit is completed in 99.34 and 99.74 nanoseconds (ns), respectively. When the interaction is weak, we can address each qubit independently by setting the frequency of an AC microwave pulse equal to the ground state Zeeman-splitting energy of each QD, which is 18.31 GHz (left) and 18.45 GHz (right) in our case. It is worth noting that the gating time as well as the driving frequency here are soundly connected to Ref.^10^. (**d**) Time responses simulated at $V_M$ = 408 mV that achieve a single-step completion of the controlled-NOT operation in 100.4 ns. A 8 mV increase of $V_M$ dramatically enhances the interaction of QDs so *J* at $V_M$ = 408 mV is $\sim$250 times larger than the case of $V_M$ = 400 mV. Once QDs strongly interact, the Rabi frequency of the qubit in one QD depends on the state of the qubit in the other QD, generating 2-qubit entanglement.

In any physical platforms, the first step for gating operations is to initialize qubits so the system can be prepared for upcoming control pulses. In the target DQD platform where a qubit state 0 and 1 are encoded to the down-spin (${|}{\downarrow}{\rangle}$) and the up-spin (${|}{\uparrow}{\rangle}$) ground state of a QD, respectively, initialization is done by manipulating biases imposed on top electrodes such that the ${|}{\downarrow}{\rangle}$ state in each QD is occupied with an electron. To quantify the range of biases that can place the target system in the (${|}{\downarrow}{\rangle}_L$, ${|}{\downarrow}{\rangle}_R$) state (= ${|}{\downarrow}{\downarrow}{\rangle}$) where the subscription *L* and *R* represent for the left and right QD, respectively, we model the charge stability with device simulations, and present the result in Fig. 3a as a function of $V_L$ and $V_R$ at $V_M$ = 400 mV, where $V_B$ is fixed to 200 mV. The stability diagram is split to 4 regimes, and each one is identified with two numbers that represent the electron population of each QD. With increasing $V_{L(R)}$, the ground state of the left(right) QD shifts down in energy and is occupied with an electron when the state touches the Fermi-level of the source electron reservoir. Establishing a strong connection to data measured for the physical DQD system^10^, our result reveals that ($V_L$, $V_R$) = (540 mV, 570 mV) (the yellow point labeled as **Pinit**) can be an initialization point that is beneficial for noise-robust qubit interactions since two QDs can be symmetrically biased^30^.

Representing the strength of inter-QD qubit interaction, the exchange energy (*J*) between two ground states serves as a source of 2-qubit entanglement in the DQD platform and can be controlled with the middle gate bias that affects the potential barrier between two QDs. In our simulations, *J* becomes 75.6 KHz at $V_M$ = 400 mV (at **Pinit**) and, as shown in Fig. 3b, sharply reaches 19.3 MHz when $V_M$ is increased by 8 mV. Changes in $V_M$ also affect Zeeman-splitting energies of the left ($E_{ZL}$) and the right ground state ($E_{ZR}$) that determine the resonance frequency of each spin qubit, but their dependence on $V_M$ is not quite noticeable such that ($E_{ZL}$, $E_{ZR}$) is (18.309 GHz, 18.453 GHz) at $V_M$ = 400 mV and changes to (18.312 GHz, 18.448 GHz) when $V_M$ is 408 mV. Due to the laterally inhomogeneous $B_Z$ (the inset of Fig. 1a), $E_{ZL}$ and $E_{ZR}$ are distinguishable and qubits can be addressed independently if their interaction is weak, and one of such cases is shown in Fig. 3c, where we simulated 2-qubit responses at $V_M$ = 400 mV with a [010]-oriented time-varying magnetic field $B_Y{(t)}$ = $B_o{cos(\omega _D t + \theta )}$ that is generated from a microwave pulse and is incorporated in modeling as elements of the Heisenberg Hamiltonian. In particular, the two subfigures here show that a $R_Y(\pi )$ operation (1-qubit rotation by $\pi$ radian around the Y-axis) can be selectively implemented with each qubit by setting $\omega _D$ to $E_{ZL}$ or $E_{ZR}$, and gating is completed in 99.34 & 99.47 ns (left & right) when $B_o$ = 5.0 MHz and $\theta$ = 0. If the interaction is not weak enough to ignore, the resonance frequency of one qubit starts to depend on the spin state ($|\downarrow \rangle$ or $|\uparrow \rangle$) of the other qubit, and a CNOT operation can be then realized with a single control pulse^10,33^. To mimic this 1-step implementation with modeling, we simulate the DQD structure at $V_M$ = 408 mV with $B_Y{(t)}$ of $\omega _D$ (= 1.832 GHz), $B_o$ (= 4.977 MHz) and $\theta$ (= 1.5$\pi$ radian) that are determined with the analytical solution driven by Russ et al.^33^. Simulated 2-qubit responses in Fig. 3d clearly show the CNOT gating is completed in 100.4 ns, being fairly connected to the experiment^10^.

**Figure 4:**
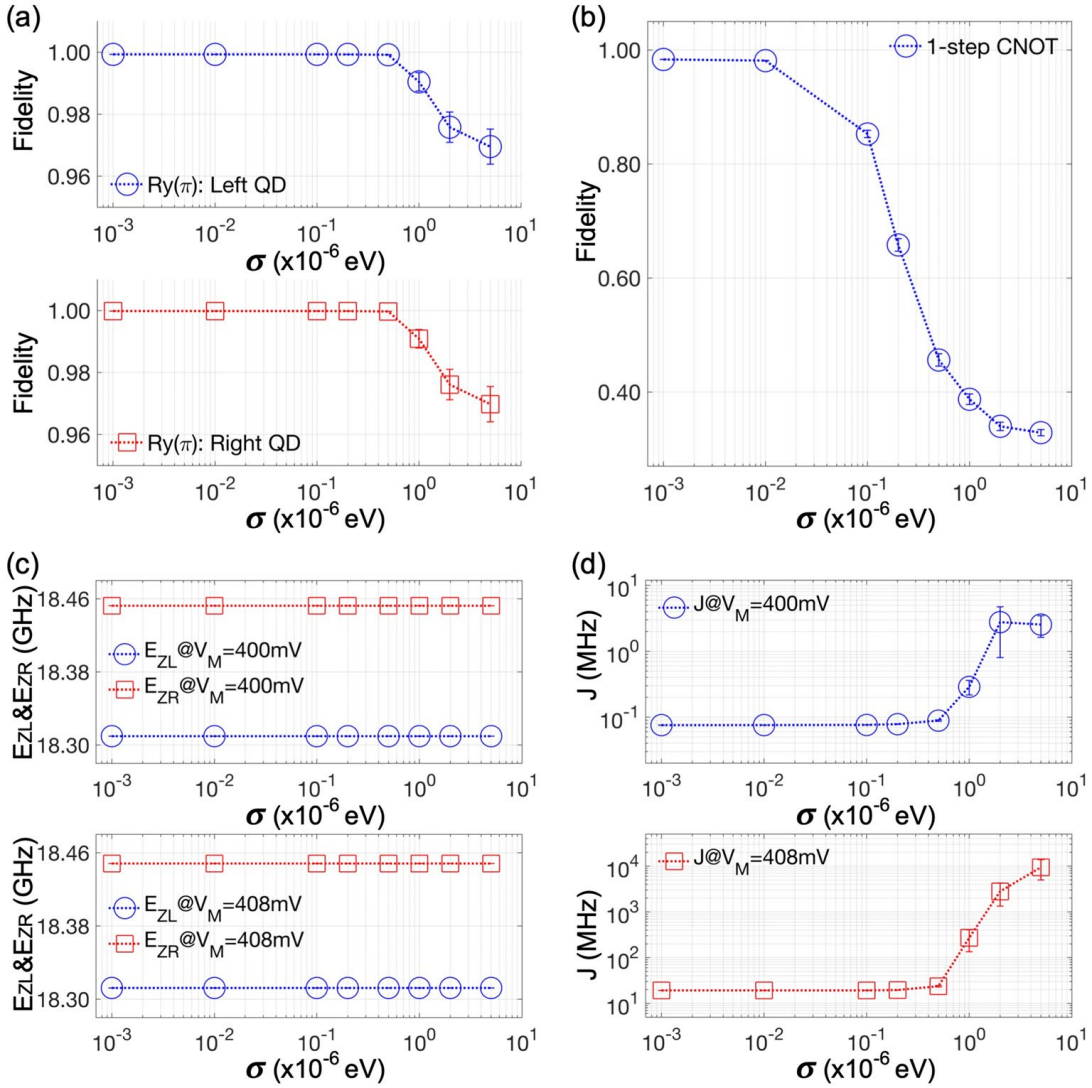
Noise-driven behaviors of 1-qubit rotation and single-step CNOT operation. (**a**) The fidelity of a $R_Y(\pi )$ operation conducted with the left and the right quantum dot (QD) are presented as a function of the magnitude of charge noise ($\sigma$: standard deviation of noisy potential values that are randomly generated per grid of the modeling domain). The fidelity obtained with 1000 samples is 99.93±10$^{-6}$% (left QD) and 99.98±10$^{-6}$% (right QD) when $\sigma$ = 10$^{-3}$ µeV, and becomes 96.95±0.5664% and 96.98±0.5687% when $\sigma$ = 5 µeV. (**b**) The single-step controlled-NOT operation turns out to be much more vulnerable to charge noise than the case of 1-qubit rotations so the fidelity becomes 98.34±0.003% and 32.84±0.5361% when $\sigma$ is 10$^{-3}$ µeV and 5 µeV, respectively. (**c**) Noise-driven fluctuation in Zeeman-splitting energy ($E_{ZL}$, $E_{ZR}$) and (**d**) exchange interaction (*J*) is shown in the regime of weak ($V_M$ = 400 mV) and strong interaction ($V_M$ = 408 mV), revealing that the degradation in fidelity, particularly in the case of entangling operation, is due to the sensitivity of *J* to charge noise.

In a noise-free condition, modeling results show that the $R_Y(\pi )$ operation is conducted for the left and the right spin with a fidelity of 99.93% and 99.98%, respectively, and the 1-step CNOT operation has a fidelity of 98.34%. To investigate how they are affected by charge noise, we simulate the system with the conditions described in the previous paragraph but disturb the clean potential profiles with random noisy values that are generated under a zero-mean gaussian distribution of a standard deviation $\sigma$. Figure 4a,b show the fidelity of the $R_Y(\pi )$ and the 1-step CNOT operation as a function of $\sigma$, respectively, where each case is modeled by conducting 1000 simulations per a single value of $\sigma$ that is varied from 10$^{-3}$ to 5 µeV. Results clearly indicate that both operations continue to lose accuracy as the DQD system experiences more severe noise, but their patterns of the noise-driven degradation in fidelity are different. In the case of $R_Y(\pi )$ gating, the fidelity turns out to be 99.93±10$^{-6}$% (left) and 99.98±10$^{-6}$% (right) at $\sigma$ = $10^{-3}$ µeV, and starts to decrease noticeably when $\sigma$ reaches 1 µeV or larger such that it drops to 96.95±0.5664% (left) and 96.97±0.5687% (right) at $\sigma$ = 5 µeV, Similarly to the $R_Y(\pi )$ case, the 1-step CNOT operation has a nice fidelity (98.34±0.003%) when $\sigma$ is $10^{-3}$ µeV. Its robustness to noise however is much worse than what $R_Y(\pi )$ shows, and the average fidelity plummets more than 60% (32.84±0.5361%) when $\sigma$ is 5 µeV.

In the extreme case where the left and right qubit never interact (*i*.*e*., *J* = 0), the Heisenberg 2-spin Hamiltonian can be completely described with Zeeman-splitting energies of spin states and external magnetic fields, and so are 2-qubit responses of the DQD system. Accordingly, in the regime of a weak interactions that can be represented with the case of $V_M$ = 400 mV, the quality of single qubit addressing under charge noise should be determined by how $E_{ZL}$ and $E_{ZR}$ behave. The origin of noise-robust $R_Y(\pi )$ rotations (Fig. 4a) can be therefore clarified with simulation results presented in the upper subfigure of Fig. 4c, which indicate that the noise-driven fluctuation in two Zeeman-splitting energies at $V_M$ = 400 mV becomes smaller than 100Hz (10$^{-5}$% of their clean values) regardless of $\sigma$. If the 2-qubit interaction is not ignorable as it is when $V_M$ = 408 mV, *J* also starts to affect the noise-robustness of gating. As Fig. 4d shows, the noise-driven fluctuation in *J* is generally much stronger than the $E_{ZL}$ & $E_{ZR}$ case, and, particularly at $V_M$ = 408 mV, it acts as the major factor that determines the noise-robustness of 2-qubit states because our results reveal that the fluctuation in $E_{ZL}$ and $E_{ZR}$ is still negligible as shown in the lower subfigure of Fig. 4c. In consequence, we can say that the huge reduction in fidelity observed in noisy 1-step CNOT operations (Fig. 4b) is mainly due to the noise-driven instability of *J*.

**Figure 5:**
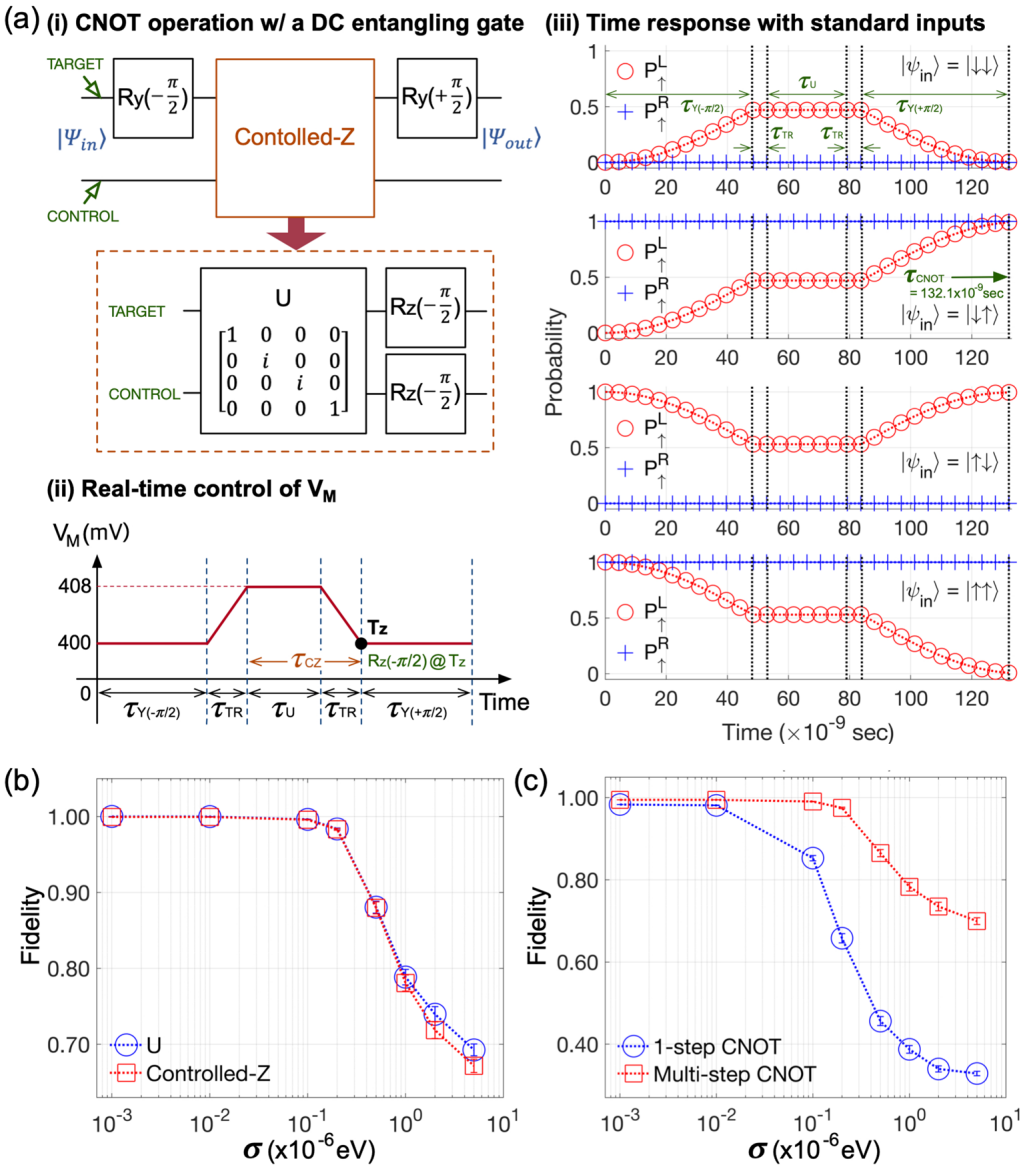
Multi-step CNOT operation with DC entangling logic. (**a**) (i) The CNOT operation can be achieved with three steps, where the second one (a controlled-Z (CZ) gate) consists of a 2-qubit unitary *U* and two 1-qubit rotation blocks ($R_Z(-\pi /2)$) that can be implemented with only DC biases in double quantum dot (DQD) platforms. Here the entanglement is generated by *U*. (ii) A real-time control of the middle gate bias ($V_M$) for the three-step CNOT operation when the left and right gate bias are 540 mV and 570 mV, respectively. A bias-transition time ($\tau _{TR}$) of 5 nanoseconds (ns) is assumed. (iii) Resulting 2-qubit time responses show that the CNOT operation is completed at 132.1 ns. (**b**) The fidelity of a *U* and a CZ block are shown as a function of the magnitude of charge noise, indicating that 1-qubit Z-rotations do not quite affect the preciseness of the overall CZ operation. (**c**) The multi-step CNOT operation is much more robust to charge noise than the single-step case, mainly due to the noise-robustness of the DC entangling block *U*.

Given that the material-inherent charge noise itself would not be easy to be eliminated or hugely suppressed, the next action for implementation of reliable CNOT operations may be to seek for engineering approaches that can make the gate more robust to “existing” noise. For this purpose, here we computationally explore one idea whose main focus is to control qubit interactions such that the “noise-sensitive” interval in time responses can be reduced as much as possible. In Fig. 5a–i, we show a simple 2-qubit circuit which also conducts a CNOT operation and will be used as a testbed of the noise-robustness. Here, the desired gating can be implemented with a time-sequential conduction of a $R_Y(-\pi /2)$, a controlled-Z (CZ), and a $R_Y(\pi /2)$ operation where $R_Y$ rotations are applied to the upper (target) qubit. Taking the right QD spin as a control qubit, we can implement the two $R_Y$ gates in the DQD platform at $V_M$ = 400 mV by setting $B_Y{(t)}$ similarly to the $R_Y(\pi )$ case except that $\theta$ is $\pi$ (instead of 0) when the rotation angle is negative. The CZ gate in the second step serves as an entangling block and can be obtained only with the DC $B_Z$ field that is generated from the micromagnet. Technically, a CZ gate can be further decomposed into 2 steps as illustrated in the bottom subfigure of Fig. 5a–i. Here, the 2-qubit controlled-phase gate *U* is *device*-*native*^33^, which means the unitary can be completely described with only DQD-native spin parameters (*i*.*e*., Zeeman-splitting energies and exchange interaction). The Z-rotation ($R_Z$) is also device-native but must be carried in the regime of a weak interaction (*e*.*g*., $V_M$ = 400 mV in our case). In real experiments, the $R_Z$ is conducted by changing the reference phase for individual spins instead of directly rotating them, which can be done conveniently with software at negligible cost in speed and accuracy^7,9,10^. In Fig. 5a-ii, we show the real-time pattern of $V_M$ that drives this multi-step CNOT gate, where $\tau _{Y}$’s and $\tau _{U}$ on the X-axis are the gating time of $R_Y(\pm \pi /2)$ and *U*, respectively. We assume that the $R_Z$ gating is performed instantaneously (at the time point labeled as $\mathbf {T_Z}$), adopting a bias-transition time ($\tau _{TR}$) of 5 ns for simulations similarly to the experiment^10^. The resulting responses in Fig. 5a-iii reveal that the entire process takes 132.1 ns, where $\tau _{Y}(\pm \pi /2)$ and $\tau _U$ become 48.1 ns and 25.9 ns, respectively.

The focal characteristic of the above-mentioned multi-step CNOT operation is that the 2-qubit entanglement is solely generated by the CZ block, and eventually by the controlled-phase unitary *U*, as all the remaining logics ($R_Y$’s and $R_Z$’s) handle 1-qubit addressing in the regime of a weak interaction. As the sensitivity of $E_{ZL}$ and $E_{ZR}$ to charge noise is not quite noticeable (Fig. 4c), the fairly nice noise-robustness of $R_Y$ shown in Fig. 4a also becomes valid for 1-qubit rotations about arbitrary axes. We can thus expect that the noise-driven fidelity of the CZ operation may strongly depend on that of *U*, and this can be confirmed with Fig. 5b that shows the simulated pattern in fidelity of CZ and *U* gating. Due to the negligible role of $R_Y$ blocks, the overall fidelity of the multi-step CNOT logic, shown with a red dotted line of square marks in Fig. 5c, also closely follows the fidelity of *U*. When $V_M$ is 408 mV, the multi-step CNOT logic generates 2-qubit entanglement in $\sim$4x less time (25.9 ns) than the 1-step gating (100.4 ns). This “reduced time-period of a strong interaction” can contribute to making the operation more robust to charge noise, so the simulated fidelity of the multi-step operation at $\sigma$ = 5 µeV becomes 69.81±0.8208% while the 1-step CNOT gate shows 32.84±0.5361% in the same conduction. Our result in Fig. 5c also confirms the core message remains effective in the entire range of $\sigma$ that is considered for simulations.

**Figure 6:**
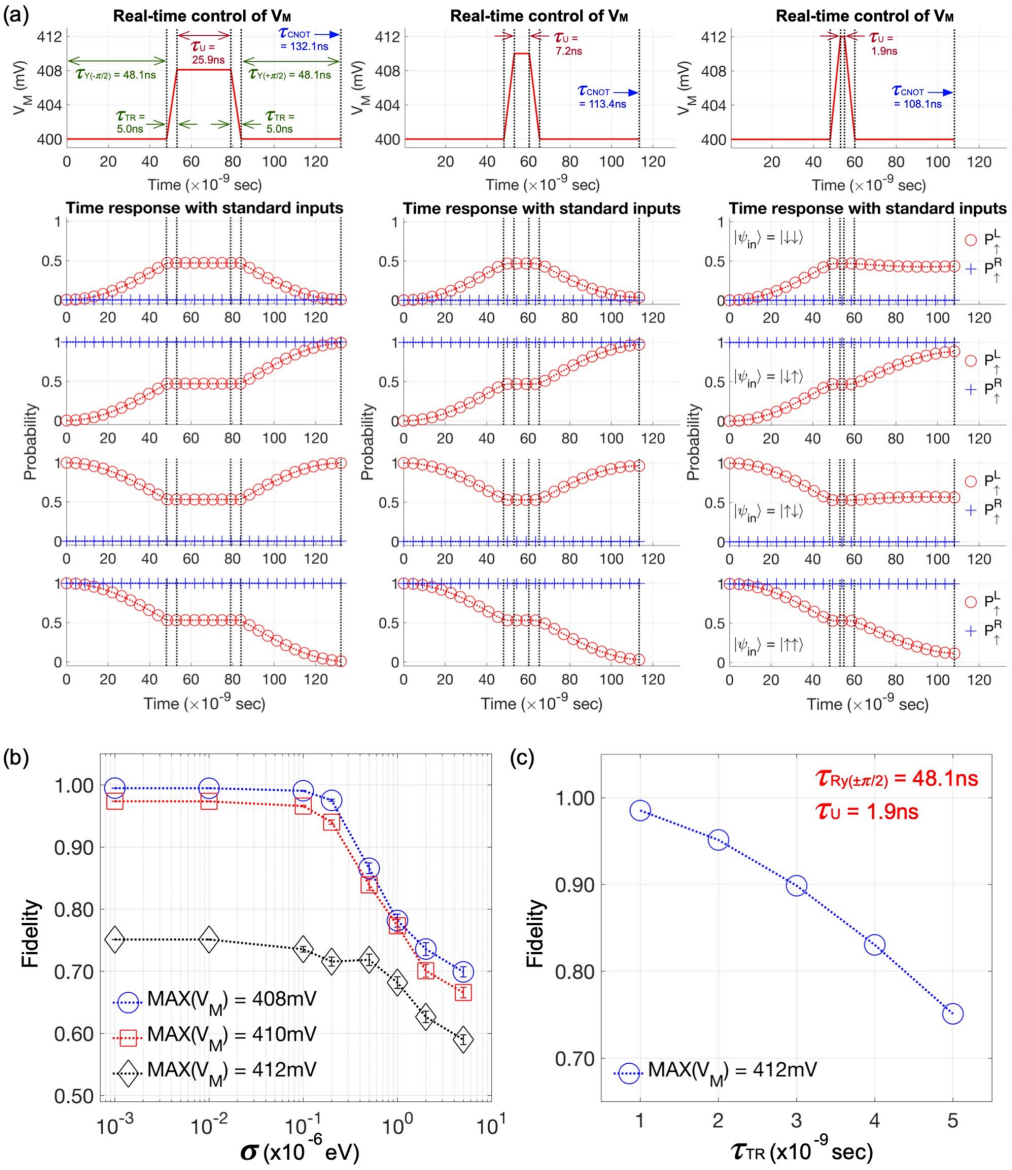
Acceleration of multi-step CNOT operation with $V_M$ control. (**a**) 2-qubit time responses are simulated for the three cases, where the middle gate bias ($V_M$) is set to 408 mV, 410 mV, and 412 mV to place the device in a regime of strong interaction. The reference case (408 mV) takes 25.9 nanoseconds (ns) to finish controlled-phase operation (*U*), and this time consumption becomes 7.2 ns and 1.9 ns when $V_M$ is 410 mV and 412 mV, respectively. (**b**) Corresponding fidelities are plotted as a function of the magnitude of charge noise, where we find the noise-driven degradation in fidelity of the second case (410 mV) does not show remarkable difference compared to the reference case though 18.7 ns can be saved for gating. The last case (412 mV) is more robust to charge noise, but the average fidelity is not good even at $\sigma$ = 10$^{-3}$ µeV (75.1%) due to the transition of $V_M$ ($\tau _{TR}$ = 5 ns), as shown in (**c**) the loss in fidelity that is calculated as a function of $\tau _{TR}$.

In Fig. 3b, we showed that the interaction energy between QDs has little effects on the resonance frequency of each spin qubit, so the gating time of *U* can be safely controlled with *J* (and thus $V_M$) with no worries for unintentional variations in any $E_{ZL}$- and $E_{ZR}$-related elements of the 4$\times$4 Heisenberg Hamiltonian^33^. With this background, we investigate what happens on the noise-robustness of the multi-step CNOT operation if the gating time of *U* is further reduced. For this purpose, the multi-step CNOT gate is simulated at $V_M$ = 410 mV and 412 mV, where other control parameters are kept the same as the previously used ones. The time responses in Fig. 6a clearly show that the entanglement is generated in 7.2 ns and 1.9 ns when $V_M$ is 410 mV and 412 mV, respectively, and thus the CNOT gating time is reduced to 113.4 ns and 108.1 ns. Figure 6b, which shows the fidelity of each noisy CNOT operation, indicates that the noise-robustness at $V_M$ = 410 mV does not quite change compared to the case of $V_M$ = 408 mV though entanglement is generated must faster (25.9 ns $\rightarrow$ 7.2 ns). This result, being different from the one obtained through a comparison between the single-step and the multi-step CNOT gate at $V_M$ = 408 mV, can be explained with the fact that the time-integration of *J* (*i*.*e*., $\int _{0}^{\tau _U} J(t) \,dt$) remains the same in the two cases (19.3 MHz$\times$25.9 ns and 69.5 MHz$\times$7.2 ns when $V_M$ is 408 mV and 410 mV, respectively), while, in the previous two cases where $V_M$ is kept the same, the time-integration becomes smaller in the multi-step operation (19.3 MHz$\times$25.9 ns) than in the single-step one (19.3 MHz$\times$100.4 ns). If $V_M$ is increased to 412 mV, the time-integration still remains similar (266.1 MHz$\times$1.9ns), showing <1% deviation from the values at $V_M$ = 408 mV and 410 mV. In this case, however, the average fidelity gets worse even under weak noise (75.1% at $\sigma$ = 10$^{-3}$ µeV), and this is due to the transition of $V_M$ that is essential to switch the interaction strength of QDs. Figure 6c, which shows the loss in fidelity of the multi-step CNOT operation at $V_M$ = 412 mV as a function of $\tau _{TR}$, indicates that the loss can be reduced with a faster bias-transition, and we observe that the fidelity is recovered back to 98.52% if the transition can be conducted in 1 ns. Overall, it is fair to say that increasing the speed of U gating has little effects on the fidelity under charge noise, but still contributes to saving the gating time, so, at $V_M$ = 410 mV where the fidelity is not yet quite affected by a 5 ns-transition of $V_M$, the multi-step CNOT gate can be completed with just 10% larger time-cost (113.4 ns) than the single-step gate (100.4 ns).

## Conclusion

Entangling logic operations under charge noise are computationally investigated in a silicon double quantum dot (DQD) system where quantum bits (qubits) are encoded to the confined electron spins. Using a realistic DQD platform based on a silicon/silicon-germanium (Si/SiGe) heterostructure, we make a solid connection to the recent experimental work^10^ where a fast controlled-X (CNOT) gate has been implemented with a single-step control, but also extend the modeling scope into noise-driven behaviors of the single-step CNOT operation and 1-qubit rotations by incorporating random noisy potential energies into device simulations. Though the 1-step implementation of a CNOT gate in the Si DQD platform has opened the fundamental pathway for securing a fast CNOT gate with simple controls, it severely suffers from charge noise due to unintended fluctuations in the interaction energy between QDs, so its fidelity reaches lower than 35% when the standard deviation of noisy potential energies ($\sigma$) is 5 µeV. In contrast, 1-qubit rotations are generally quite robust to charge noise since the noisy fluctuation in potential distributions hardly affects the resonance frequency of individual spins. Employing a DQD-native controlled-phase operation can be remarkably helpful for noise-robust implementation of a CNOT gate, because, at the same strength of 2-qubit interaction, it generates quantum entanglement much faster than the single-step CNOT operation. Although additional 1-qubit rotations need to be conducted sequentially in time to complete the CNOT operation, they have little effects on the noise-robustness, so the overall fidelity reaches $\sim$70% at $\sigma$= 5 µeV in spite of the increased complexity in device controls associated with additional 1-qubit rotations. Another benefit of the controlled-phase operation implemented in the DQD platform is that its speed can be enhanced by increasing the strength of 2-qubit interaction with almost no degradation in noise-robustness. In consequence, the associated CNOT gating can be conducted as fast as the single-step operation. Being supported with rigorous simulations, the engineering details discussed in this work can contribute to elevating the current status of a Si QD platform for robust designs of scalable quantum processors. Finally, we remark that it would be worth investigating the possibility for extending this work to designs of multi-qubit controlled nonadiabatic holonomic gates^44,45^, which may also contribute to increasing the noise-robustness of entangling operations.

## Data Availability

The datasets generated and analysed during the current study are available from the corresponding author on reasonable request.
